# The professionalism of psychiatry registrars as perceived by patients and various health practitioners at Weskoppies Psychiatric Hospital, Pretoria

**DOI:** 10.4102/sajpsychiatry.v24i0.1166

**Published:** 2018-04-10

**Authors:** Matthews M. Banda, Werdie C.W. van Staden

**Affiliations:** 1 Department of Psychiatry, University of Pretoria, South Africa

## Abstract

**Objectives:**

Amidst calls for improved professionalism, this study examined the professionalism of psychiatry registrars at Weskoppies Hospital as evaluated by their patients, themselves, their consultants and other health practitioners. The second objective was to examine the perceived importance of aspects of professionalism and compare these descriptively among the various health practitioners and patients.

**Method:**

Participants completed the Professionalism Mini-Evaluation Exercise Questionnaire in evaluating the professionalism of the registrar. The number of questionnaires completed by patients, allied health practitioners, consultant psychiatrists and psychiatry registrars were, respectively, 100, 50, 25 and 20; thus, 195 in total. This previously validated questionnaire consists of 21 items that enquire about doctor-patient relationship skills, reflective skills, time management and interprofessional relationship skills. Participants also ranked the three items of the questionnaire that they considered most important.

**Results:**

Highly statistically significant differences among four groups emerged for both the total and all four subscale scores, with patients generally rating the professionalism of registrars as lower, and not meeting with expectations. All four groups ranked ‘listened actively to patient’ and ‘showed interest in patient as person’ highly. Patients and allied health practitioners ranked the attribute ‘was on time’ highly, while consultants and registrars did not.

**Conclusion:**

Improving professionalism requires that the attributes ‘listening actively to patient’ and ‘recognising and meeting patient needs’ are taken seriously. Doing so requires that patients also evaluate the professionalism of registrars, rather than relying merely on the evaluation by consultants as being sufficient.

## Introduction

Professionalism in medical practice is unequivocally considered a virtuous pursuit, as captured, for example, in the Charter on Medical Professionalsim.^1,2^ Compared to the professionalism of other medical colleagues, psychiatrists may generally be held to a higher standard owing to the nature of the relationship with their patients, the private and intimate information to which they are privy, the challenges of treating people against their will, as well as the challenging if not eroding effects that a mental disorder itself has on professionalism at times.^3,4^

In South Africa, no research has yet been performed on the professionalism of psychiatrists or psychiatric registrars. Elsewhere, professionalism has been evaluated through various instruments,^5^ but hardly any studies have examined professionalism specifically in psychiatry even though this has been highlighted as important.^2,3^ Exceptions include a study on multisource feedback (MSF) regarding psychiatrists’ communication skills, professionalism, collegiality and self-management.^6^ Professionalism also features in a study that uses MSF for assessing trainees in child and adolescent psychiatry.^7^

Distinct from measuring professionalism in psychiatry, it has been identified as a training need. Psychiatry trainees were evaluated in North-West England through an online survey for meeting standards of professionalism education.^8^ Trainees considered professionalism education as important, yet standards of professionalism education were generally not met. They lacked formal teaching and educational opportunities, and 20% of supervisors were considered to be ‘not good’ role models. Similar opinions were expressed by 151 psychiatry residents across seven psychiatry residency programmes in the United States who strongly supported a curriculum in professionalism and ethics and valued its relevance in the practice of psychiatry.^9^ Psychiatry residents in seven other programmes in the United States were surveyed about their views and preferences regarding various methods of assessment of professionalism.^10^ Three significant findings emerged: They strongly agreed that clinical supervision was an appropriate assessment method; they found clinical supervision to be better than oral examinations, short answer questions, essays or standardised patient interactions; and they strongly favoured direct faculty observation of their interactions with actual patients and clinical team members.

Various ways have been emphasised to maximise professionalism in psychiatry even before specialising in psychiatry.^11^ Bughra underscores that the selection of students at both undergraduate and postgraduate level is crucial in developing medical professionalism in the 21st century, as some attitudes can be changed through learning and mentoring, whereas others cannot and will not.^11^ Learning and mentoring of professionalism in psychiatry begin already during undergraduate years and should be maintained and developed even for the seasoned practitioner through, for example, continued professional development events.

Teaching of professionalism may progress from didactic education, supervised training, indirect supervision to eventually it being practiced autonomously.^12^ This may be an overt objective as required by the Accreditation Council for Graduate Medical Education in the United States as one of the six core competencies,^13^ yet often remains an afterthought in the standardised registrar/residency curriculum, being rather part of the ‘hidden curriculum’.^3,14^ Accordingly, rather than through teaching, learning of professionalism may result mostly from role models through the observation of supervisors and mentors in their professional conduct (whether good or bad).^11,15,16,17^

Professionalism is usually judged by peer review, but this may be misleading as seen in study among medical students and residents who rated their own professionalism, the other groups’, as well as their faculty members’ professionalism.^18^ Both groups of medical students on one hand and residents on the other hand viewed their peer groups as more professional than the other. In addition, both medical students and residents rated faculty members as the poorest in terms of observed professional behaviours. These differences suggest that MSF may be a better approach to assess professionalism of registrars.^6,7^

Considering the lack of research on professionalism in psychiatry, and no prior studies on this in South Africa, our study examined the professionalism of psychiatry registrars in a local, discipline-specific context. As suggested by MSF and is crucial in a person-centred approach,^19,20^ patients should have a say in the matter. Hence, this study examined the professionalism of registrars as evaluated by their patients, themselves, their consultants and other health practitioners. The second objective was to examine the perceived importance of aspects of professionalism in psychiatric care and compare these descriptively among the various health practitioners and patients, for this has neither been reported in the literature before.

## Methods

### Design and setting

This quantitative cross-sectional study was conducted at Weskoppies Psychiatric Hospital in Pretoria that renders tertiary and quaternary inpatient and outpatient psychiatric services. Tertiary in this context means the hospital provides for patients whose healthcare needs could not be met at primary or secondary public healthcare services or in the private sector. Quaternary services mean that patients are referred for highly specialised psychiatric care. This site is affiliated to the University of Pretoria where psychiatry registrars do their clinical training. The study received ethics approval from the Faculty of Health Sciences Research Ethics Committee at the University of Pretoria – approval number 366/2014. The Weskoppies Hospital’s chief executive officer gave permission for the study to be conducted among the participants on the hospital premises.

### Participants

Participants included patients, psychiatry registrars, psychiatry consultants supervising the specific registrars and various other health practitioners who worked in the same interdisciplinary team as the particular registrar whose professionalism they evaluated. Criteria for the inclusion of patients included being an adult aged at least 18 years who had used the psychiatry inpatient and/or outpatient services of Weskoppies Hospital during the preceding 3 months and were able to comprehend and complete the Professionalism Mini-Evaluation Exercise Questionnaire (P-MEX). A validated translation of the P-MEX was not available. As the P-MEX was submitted anonymously, participants were not assisted by an interpreter or otherwise. Each patient was required to evaluate the registrar who had mainly been treating him or her in the preceding 2 months. Psychiatry consultants evaluated individually each of the one to two registrars who they had been supervising at that time. The allied health practitioners comprised nursing personnel, occupational therapists, psychologists and social workers who evaluated the registrars who had been working in their interdisciplinary team during the preceding 2 months. The registrars had to evaluate their own professionalism.

All participants gave informed consent before participating in the study, and they submitted the questionnaires anonymously. As explained in the informed consent document, the questionnaire was headed by an instruction as to evaluate a specific registrar and return the questionnaire anonymously at a dedicated collection point. As the questionnaires solicited sensitive and potentially harmful information, their anonymous submission was considered important and no demographic data were collected so as to prevent the anonymity of participants being undermined, particularly among the consultants, registrars and other health practitioners who were relatively few in numbers.

The total sample size was 195. Patients had been recruited and sampled conveniently until 100 were included. The number of questionnaires completed and returned by allied health practitioners, consultant psychiatrist and registrars were, respectively, 50, 25 and 20. The 20 registrars who returned their questionnaires constituted 91% of the 22 psychiatry registrars in clinical training at the University of Pretoria at the time

### Measuring instrument

The P-MEX is a validated instrument designed to assess and evaluate professionalism.^21^ The questionnaire was first validated at McGill University on students rotating in various clinical specialties. The questionnaire has since been used in various settings and countries, and found to be a valid tool in assessing professionalism.^22,23,24,25^ The questionnaire consists of 21 items, each to be rated as not acceptable, below expectation, met expectation and exceeded expectation. The 21 items measure various aspects of professionalism comprising the following statistically derived subscales: skills in doctor-patient relationship, reflective skills, time management and interprofessional relationship skills. The subscales were not labelled as such on the questionnaire that participants completed.

After rating registrars on the questionnaire, participants selected and ranked the three most important items among the 21, in order of importance to them.

### Analyses

Ratings for each item were scored from 1 to 4 matching the categories from ‘unacceptable’ to ‘exceeded expectations’. Non-parametric tests were used for statistical comparisons considering the smaller sample sizes of the registrar and the consultant groups and that a normal distribution should not be assumed. The four groups were compared statistically for each item of the questionnaire using the Fisher’s Exact Test. Subscales were compared across all four groups using the Kruskal-Wallis test; and post hoc analyses compared the patient group to each of the other groups using the Mann-Whitney U test (MWU). Internal consistency among items of each of the subscales was tested by calculating Cronbach’s alpha coefficients.

The highest ranked item was assigned a value of 3, the second ranked item was assigned a value of 2 and the third ranked item was assigned a value of 1. For purposes of standardisation across groups of different sizes, summations of these values for each item were expressed as a fraction of 100 (i.e. a percentage) for each group in order to compare descriptively the rankings of items among the participant groups. Thus, the ratio of the cumulative scored rankings between a particular item and all items was expressed as a fraction of 100. This was done for each group and across all participants.

## Ethical consideration

All participants gave informed consent before participating in the study, and they submitted the questionnaires anonymously. The study received ethics approval from the Faculty of Health Sciences Research Ethics Committee at the University of Pretoria – approval number 366/2014. The Weskoppies Hospital chief executive officer gave permission for the study to be conducted among the participants on the hospital premises.

## Results

Table 1 shows the average frequency of the four categories of evaluations on the 21 items of the P-MEX, expressed as a proportion for each of the participant groups.

**TABLE 1:** Average frequencies of ratings by participant group (expressed as a percentage).

Variable	Proportion of patients *n* = 100	Proportion of registrars *n* = 20	Proportions of consultants *n* = 25	Proportions of allied health practitioners *n* = 50
Not acceptable (%)	1	0	0	0
Below expectations (%)	54	5	4	21
Met expectations (%)	38	65	72	60
Exceeded expectations (%)	7	30	24	19

Table 2 shows the means and their standard deviations for the scored ratings of the four groups. Mean scores above 3 indicate that professionalism expectations were met or exceeded, whereas those below 3 indicate that registrars were considered to be below professionalism expectations. As seen in Tables 1 and 2, the professionalism of registrars was rated similarly by the consultants and the registrars (who rated their own professionalism) as on average meeting or exceeding expectations. The allied health professional rated the professionalism of registrars lower but not as low as the patients did who on average considered registrars not meeting expectations on all of the subscales.

**TABLE 2:** Comparisons among the four participant groups for the scaled scores on the subscales of the Professionalism Mini-Evaluation Exercise Questionnaire.

Variable	Means and standard deviations								Kruskal-Wallis value (df = 3)	Statistical significance
	Patients *n* = 100 (%)		Registrars *n* = 20 (%)		Consultants *n* = 25 (%)		Allied health professionals *n* = 50 (%)			
Doctor-patient skills	2.59	0.35	3.28	0.32	3.25	0.45	2.93	0.43	71.14	*p* < 0.0001
Reflective skills	2.57	0.39	3.19	0.41	3.15	0.46	2.93	0.46	55.70	*p* < 0.0001
Time management	2.62	0.44	3.15	0.50	3.24	0.64	2.96	0.41	45.09	*p* < 0.0001
Interprofessional skills	2.61	0.39	3.33	0.26	3.35	0.57	3.0	0.40	74.86	*p* < 0.0001

The four groups were statistically significantly different for each of the items (for each item, *p* < 0.0001 using Fisher’s Exact Test). Each of the subscales was statistically significant across all four groups, as shown in Table 2. In the post hoc analyses, the patients evaluated registrars statistically significantly lower than the other groups for all subscales, as follows: Regarding doctor-patient skills, the patient group was highly statistically significantly different from registrars (MWU = 132; *p* < 0.0001), consultants (MWU = 290.5; *p* < 0.0001) and allied health practitioners (MWU = 1329; *p* < 0.0001). Regarding reflective skills, the patient group was highly statistically significantly different from registrars (MWU = 280.0; *p* < 0.0001), consultants (MWU = 361.5; *p* < 0.0001) and allied health practitioners (MWU = 1369.5; *p* < 0.0001). Regarding management skills, the patient group was highly statistically significantly different from registrars (MWU = 437.0; *p* < 0.0001), consultants (MWU = 412.0; *p* < 0.0001) and allied health practitioners (MWU = 1481.5; *p* < 0.0001). Regarding interprofessional skills, the patient group was highly statistically significantly different from registrars (MWU = 125; *p* < 0.0001), consultants (MWU = 306.5; *p* < 0.0001) and allied health practitioners (MWU = 1187.5; *p* < 0.0001).

As an indication of internal reliability, the items measured consistently with good Cronbach’s alpha coefficients for the items measuring doctor-patient skills (0.78), reflective skills (0.75), interprofessional skills (0.84) and fair consistency for the time management items (0.59).

Regarding participants’ ranking of the professionalism items they considered most important, Table 3 lists the top five ranked items by participant group. Table 4 shows the standardised scores of the rankings by the four subscales of the P-MEX.

**TABLE 3:** Ranking of items by participants (with standardised scoring of rankings in %).

Variable	Patients	Allied health practitioners	Consultants	Registrars
Highest ranked item	Listened actively to patient (19%)	Listened actively to patient (18%)	Recognised and met patient needs (21%)	Listened actively to patient (27%)
Second highest ranked item	Admitted errors or omissions (11%)	Ensured continuity of patient care (15%)	Listened actively to patient (15%)	Recognised and met patient needs (23%)
Third highest ranked item	Recognised and met patient needs (9%)	Showed interest in patient as person (11%)	Showed interest in patient as a person (14%)	Ensured continuity of patient care (9%)
Fourth highest ranked item	Was on time (9%)	Admitted errors or omissions (10%)	Advocated on behalf of patient (11%)	Showed interest in patient as a person (8%)
Fifth highest ranked item	Showed interest in patient as a person (8%)	Was on time (7%)	Ensured continuity of patient care (8%)	Addressed own gaps in knowledge and skills (7%)
Other items	44%	39%	31%	26%

**Total of fractions by column**	**100%**	**100%**	**100%**	**100%**

**TABLE 4:** Standardised scores for rankings of professionalism items by the subscales of the Professionalism Mini-Evaluation Exercise Questionnaire.

Subscale of the P-MEX	Registrars	Consultants	Patients	Allied health practitioners
Doctor-patient relationship skills	71.7	73.5	52.0	57.0
Reflective skills (%)	9.2	14.4	32.8	24.7
Time management (%)	6.6	0.8	9.0	9.3
Interprofessional skills (%)	12.5	11.3	6.2	9.0

**Total (%)**	**100.0**	**100.0**	**100.0**	**100.0**

## Discussion

The main findings of the study were that patients rated the professionalism of their attending psychiatry registrar statistically significantly lower than did allied health practitioners, psychiatry consultants and psychiatry registrars. The attributes ‘listened actively to patient’ and ‘showed interest in patient as a person’ were ranked highly by all four groups. The attributes that were highly ranked by patients and allied health practitioners, and not by consultants or registrars, were ‘was on time’ and ‘admitted errors or omissions’. All four participant groups emphasised doctor-patient skills more than reflective, time management and interprofessional skills, but registrars and consultants emphasised these more so than patients and allied health practitioners. Reflective skills were emphasised by patients and allied health practitioners more so than the registrars and consultants.

These are the first reported findings on the professionalism of psychiatry registrars in a local, discipline-specific context. The differences among the four participant groups in, first, the ratings on the performance of registrars and, second, the differences in rankings of importance of professionalism items, underscore the importance of MSF advocated in previous studies.^6,7^

Whilst the professionalism of registrars in psychiatry was rated similarly between registrars and consultants as generally meeting or exceeding expectations, patients indicated in general that registrars’ professionalism was below expectations. This cautions against taking the consultants’ view – that is the view of the trainers of professionalism in psychiatry – as sufficient in evaluating the professionalism of registrars. With data lacking at other settings where registrars in psychiatry are trained, consultants who suppose similarly that the professionalism of their registrars is meeting with expectations of patients may be misguided. The implication for supervision is that consultants should not only rely on their own views regarding a registrar’s professionalism but also obtain views from patients and other health practitioners. Incorporating multiple views on professionalism in supervision is yet to be researched. Doing so will contribute to an evidence base for effective psychiatric supervision, which is rather undeveloped at this time.^26^

The difference between the views of patients and the other participants emerged not only regarding registrars meeting expectations of professionalism but also regarding which professionalism attributes were considered most important. These differences raise the question on how professionalism may be improved, particularly from patients’ point of view – a question that is relevant for each psychiatrist’s practice, further research and the training of registrars in professionalism.

A good point of departure in addressing this question in all three domains of practice, further research and registrar training is suggested by the concurrence of all participants on the most important professionalism skills: listening actively to patient and recognising and meeting patient needs. That is, if we want to improve professionalism, we need to recognise and meet the patient’s needs in improving professionalism and we need to listen to the patient on how to do so. In practice, this should happen within the doctor-patient relationship drawing on reflective skills that underpin (in part) professionalism. Similarly, for further research, which should involve patients on how professionalism may be improved and skills to this end may be developed.

In the clinical training of registrars, by this point of departure, patients should be engaged on the professionalism of the registrar rather than merely rely on the feedback to the registrar by the consultant who supervises and trains him or her. Multisource-guided feedback from patients, co-workers and psychiatrist colleagues on competencies in professionalism, communication skills collegiality and self-management through a five-point Likert-scale was shown to be feasible in a study by Violato et al.^6^ Team supervision has congruently been advocated in the work by Keshavan.^12^ Drawing on multiple sources in the contribution to and evaluation of professionalism of psychiatry registrars gives effect to the ethics of person-centred psychiatry and taking diversity of values seriously.^27,28,29^

At a policy level, our results suggest that clinical training of registrars in professionalism may need to be revisited at the various training sites as well as the provisions for this in the curriculum as examined by the College of Psychiatrists. Some provisions in this regard are well-established, for example, the regular FCPsych Part II questions on psychiatric ethics, but challenges in assessing attitudes and actual patient interactions remain, as acknowledged elsewhere.^30,31^

Specific aspects of professionalism that registrars ranked lower than patients relate to time management and admission to mistakes and omissions. Being punctual, for example, might have been more important to patients than registrars thought. Institutional arrangements pertaining to the setting of our study whereby patients were seen on a first-come-first-seen basis in spite of a booking for that day might have influenced patient perceptions. Other influences might have been, for example, the patient’s mood. These influences, nevertheless, point back to professionalism by which these should be addressed with the patient and/or the institution as a matter of policy.

The results of our study pertain to a particular setting and may be different at other training settings of psychiatry registrars in South Africa. Similar studies at other sites may allow for generalisation of our results, but without multisource data at the other sites, the extent of professionalism there would be at best is a matter of speculation. The results were further limited to participants who returned the questionnaires and were able to complete the questionnaire in English. Although all the practitioners and almost all patients attending at this setting were able to understand and speak English, the findings should not be assumed as accurate for those so excluded without further research using a validated translation of the P-MEX. A further limitation to our study is the instrument used to evaluate professionalism. Although the P-MEX validity is well-established and it compares well with other instruments,^5,21^ it is seemingly not exhaustive of all aspects that are authentically attributed to professionalism. Consider, for example, the Physician Charter that is widely used in defining medical professionalism.^1,2^ The charter consists of three fundamental principles of professionalism and ten professional responsibilities. The three fundamental principles are primacy of patient welfare, patient autonomy and social justice. The 10 responsibilities are commitments to professional competence, honesty with patients, patient confidentiality, maintaining appropriate relations, improving quality of care, improving access to care, a just distribution of finite resources, scientific knowledge, maintaining trust by managing conflicts of interest and professional responsibilities. Several of these aspects are not covered by the P-MEX.

As neither the professionalism of psychiatry registrars nor their clinical training is a static matter, but ideally continuously developing, a longitudinal study that tracks the development of professionalism during their training period should be more informative than our cross-sectional study. Probably even better would be to design a randomised controlled trial for the investigation of specific interventions to improve and develop psychiatry registrars’ professionalism.

Prevailing nonetheless, in fostering increasing professionalism, we need to recognise and praise the excellent role model in professionalism as and when he or she acts accordingly, for their laudable professional qualities and actions disappear easily in a study like this which concerns the general rather than the individual case.
